# Generalised Osteoarthritis Exacerbates Pain and Disability in Hand Osteoarthritis: A Single-Centre Retrospective Study

**DOI:** 10.31138/mjr.150625.dao

**Published:** 2025-09-20

**Authors:** Veronika Farská, Ivan Rybár

**Affiliations:** Department of Rheumatology, Medical Faculty of Slovak Medical University and National Institute of Rheumatic Diseases, Piešťany, Slovakia

**Keywords:** osteoarthritis, hand, pain measurement, disability evaluation, epidemiology, retrospective studies

## Abstract

**Background:**

Hand osteoarthritis (HOA) is common in older adults and often coexists with osteoarthritis (OA) in other joints.

**Objective:**

To determine whether HOA occurs in isolation or as part of generalised OA, and to assess the impact of coexisting joint involvement on pain and functional outcomes.

**Materials and Methods:**

This retrospective study included 75 patients with radiographically confirmed HOA seen between January and June 2019. Patients were classified as having isolated HOA (n = 31) or generalised OA (n = 44). Pain and function were assessed using VAS, FIHOA, and HAQ. Non-parametric tests with p-values and 95% confidence intervals were used.

**Results:**

Isolated HOA was present in 41% of patients, while 59% had OA in additional joints—most commonly the knees (68%), spine (55%), and hips (32%). Patients with generalised OA reported slightly higher pain (median VAS 61 mm vs. 61 mm; difference 0 mm, 95% CI: –26 to 34; p = 0.55) and greater functional impairment (median HAQ 1.0 vs. 0.75; difference 0.25, 95% CI: 0.0 to 0.5; p = 0.10), though differences were not statistically significant. Significant positive correlations were observed between FIHOA, VAS, and HAQ, strongest between FIHOA and VAS in generalised OA (rho = 0.75, 95% CI: 0.58 to 0.86; p < 0.001).

**Conclusion:**

HOA frequently coexists with OA in other joints and may contribute to greater pain and disability, highlighting the need for comprehensive assessment.

## INTRODUCTION

Hand osteoarthritis (HOA) is one of the most prevalent musculoskeletal conditions, particularly among older adults, with a reported radiographic prevalence exceeding 50% in individuals over 60 years of age.^1^ Although it is commonly considered a localised form of osteoarthritis (OA), increasing evidence suggests that HOA may often coexist with OA in other joint sites, such as the knees, hips, and spine.^2–4^ The clinical impact of HOA extends beyond radiographic findings. It can significantly impair hand function, reduce grip strength, and compromise performance in daily activities.^5,6^ Instruments such as the Functional Index for Hand Osteoarthritis (FIHOA)^7^ and the Health Assessment Questionnaire (HAQ)^8^ are widely used to assess function and disability in this patient population. However, these instruments typically focus on the hand alone, potentially overlooking the influence of more widespread joint involvement on functional outcomes. Generalised OA is increasingly recognised as a systemic disease with biomechanical, metabolic, and inflammatory components that may affect multiple joints.^9^ Yet, the extent to which OA in non-hand joints (e.g., knees, hips, spine) contributes to the pain and disability attributed to HOA remains insufficiently explored. This study aims to determine whether HOA predominantly occurs as an isolated condition or is more commonly associated with osteoarthritis in other joint regions. We also investigate whether the presence of OA outside the hands contributes to increased functional impairment and pain. Specifically, we compare the functional status and pain levels between patients with isolated HOA and those with HOA accompanied by osteoarthritis in other joints, such as the knees, hips, or spine.

## MATERIALS AND METHODS

### Study Design and Setting

This retrospective observational study was conducted at the National Institute of Rheumatic Diseases, Piešťany, Slovakia, among patients who attended the Rheumatology outpatient clinic between January 1, 2019, and June 30, 2019. The study received ethical approval from the Institutional Ethics Committee of the National Institute of Rheumatic Diseases, Piešťany, Slovakia, on May 16, 2018 (Project number NURCH/2018/05).

### Study Population and Sample Size

No formal sample size calculation was performed. The sample included all eligible patients meeting inclusion criteria within the specified study period.

We retrospectively analysed a consecutive series of 75 patients with radiographically confirmed hand osteoarthritis. Based on the presence of osteoarthritis in other joints, patients were divided into two groups: isolated hand osteoarthritis and generalised osteoarthritis. Pain was measured using a visual analog scale. Functional status was assessed with patient-reported outcome measures. Statistical comparisons between groups were performed using non-parametric and categorical tests with a significance level of p < 0.05.

### Inclusion and Exclusion Criteria

Inclusion criteria were as follows:

- Age ≥ 40 years;
- Clinical diagnosis of hand osteoarthritis according to the American College of Rheumatology (ACR) criteria;
- Radiographic evidence of OA in at least one hand joint (DIP, PIP, MCP, or CMC) with a Kellgren–Lawrence^9^ grade ≥ 2;
- Availability of complete clinical data, including pain assessment, functional indices, and comorbidity evaluation.
- Patients with inflammatory arthritis (e.g., rheumatoid arthritis, psoriatic arthritis), prior joint surgery in the hands, or other systemic musculoskeletal conditions were excluded.

Group A comprised patients with isolated HOA, defined as OA limited to the small joints of the hand—namely the distal interphalangeal (DIP), proximal interphalangeal (PIP), metacarpophalangeal (MCP), or first carpometacarpal (CMC) joints, based on clinical and/or radiographic findings. Group B included patients with HOA and additional OA in other joints, such as the knees, hips, or spine, determined by clinical evaluation and patient history.

### Variables and Outcome Measures

The primary outcome measures included pain intensity assessed by a 100 mm visual analog scale (VAS), functional status measured by the Functional Index for Hand Osteoarthritis (FIHOA) and the Health Assessment Questionnaire (HAQ). Additional variables included demographic data (age, sex, body mass index) and the presence of osteoarthritis in other joints based on clinical evaluation.

### Bias

As a retrospective analysis of consecutively selected patients, selection bias was minimised. However, information bias could not be entirely excluded due to reliance on medical records for some variables.

### Statistical Analysis

All statistical analyses were performed using IBM SPSS Statistics, Version 26 (IBM Corp., Armonk, NY, USA). The distribution of continuous variables—including age, body mass index (BMI), pain intensity (VAS), FIHOA, and HAQ—was assessed using the Shapiro–Wilk test within each study group (Isolated HOA: n = 31; Generalised OA: n = 44). BMI in the generalised OA group (p = 0.0314) and VAS in both groups (p = 0.0195 and p = 0.0027, respectively) did not meet the criteria for normal distribution (all p-values < 0.05). Consequently, all continuous data are reported as median and range (Min–Max), and between-group comparisons were performed using the Mann–Whitney U test. Categorical variables were analysed using the chi-square (χ²) test, with both the test statistic and p-values reported. Correlations between pain intensity (VAS), hand-specific functional status (FIHOA), and global functional status (HAQ) were assessed using Spearman’s rank correlation coefficient within each group. A two-tailed p-value < 0.05 was considered statistically significant.

## RESULTS

A total of 75 patients with HOA were included in the analysis, of whom 31 (41.3%) had isolated HOA and 44 (58.7%) had concomitant OA in other joints (generalised OA group).

The main demographic characteristics of the study population are presented in **Table 1**. In the isolated HOA group, 74,2% were female, while in the generalised OA group, 90.9% were female, confirming a predominance of women in both categories. The proportion of female patients did not differ significantly between groups (Isolated HOA: 74.2% vs. Generalised OA: 90.9%; χ^2^ = 2.64, p = 0.10).

**Table 1.** Patient characteristics by group.

**Variable**	**Isolated HOA (n=31) Median (Min–Max) or %**	**Generalised OA (n=44) Median (Min–Max) or %**	**Median Difference (95% CI)**	**p-value**
Age (years)	62.0 (36–81)	63.0 (41–87)	1.0 (–4 to 7)	0.63
BMI	25.8 (20.3–34.0)	26.4 (20.2–38.9)	0.6 (–1.0 to 2.3)	0.42
Pain VAS (mm)	61.0 (6–96)	61.0 (12–95)	0.0 (–26 to 34)	0.62
FIHOA	1.10 (0.0–2.6)	1.10 (0.1–2.3)	0.0 (–0.4 to 0.4)	0.99
HAQ	0.75 (0.12–2.0)	1.00 (0.25–2.5)	0.25 (0.0 to 0.5)	0.10
Female (%)	74,2%	90.9%	—	χ^2^ = 2.64; p = 0.10

Comparison of demographic and clinical characteristics between patients with isolated hand osteoarthritis (HOA) and those with generalised OA. Continuous variables are presented as median and range (Min–Max), with median differences and corresponding 95% confidence intervals (CI). Categorical data are presented as percentages, with chi-square (χ^2^) values and p-values. P-values were calculated using the Mann–Whitney U test for continuous variables and the chi-square test for categorical variables.

VAS: visual analog scale; FIHOA: Functional Index for Hand Osteoarthritis; HAQ: Health Assessment Questionnaire; BMI: body mass index.

The median age was 62 years (range 36–81) in the isolated HOA group and 63 years (range 41–87) in the generalised OA group (median difference 1 year, 95% CI: –4 to 7; p = 0.63). The median BMI was 25.8 (range 20.3–34.0) in the isolated group and 26.4 (range 20.2–38.9) in the generalised group (median difference 0.6, 95% CI: –1.0 to 2.3; p = 0.42). There were no statistically significant differences between the isolated HOA and generalised OA groups in age, BMI, pain VAS, FIHOA, HAQ, or the proportion of female patients.

In the generalised OA subgroup (n = 44), the most frequently involved additional joints were the knees, spine, and hips (**Table 2**).

**Table 2.** Distribution of additional joint osteoarthritis among patients with generalised OA.

**Joint**	**n (out of 44)**	**%**
Knees	30	68.2
Spine	24	54.5
Hips	14	31.8

Frequency and percentage of osteoarthritis involvement in non-hand joints among patients classified with generalised osteoarthritis (OA). Data represent the number and proportion of patients (n = 44) with OA in each joint region.

**Table 3.** Comparison of outcomes by OA Group.

**Variable**	**Isolated HOA Median (Min–Max)**	**Generalised OA Median (Min–Max)**	**Median Difference (95% CI)**	**p-value**
VAS (mm)	61.0 (6–96)	61.0 (12–95)	0.0 (–26 to 34)	0.62
FIHOA	1.10 (0.0–2.6)	1.10 (0.1–2.3)	0.0 (–0.4 to 0.4)	0.99
HAQ	0.75 (0.12–2.0)	1.00 (0.25–2.5)	0.25 (0.0 to 0.5)	0.10

Comparison of pain and functional outcomes between patients with isolated hand osteoarthritis (HOA) and those with generalised osteoarthritis

(OA). Values are expressed as median and range (Min–Max). Median differences with corresponding 95% confidence intervals (CI) are presented.

P-values were calculated using the Mann–Whitney U test.

VAS: visual analog scale; FIHOA: Functional Index for Hand Osteoarthritis; HAQ: Health Assessment Questionnaire.

Patients with generalised OA reported higher levels of pain and disability, although the differences did not reach statistical significance. The median VAS score was 61 mm (range 6–96) in the isolated HOA group and 61 mm (range 12–95) in the generalised group (median difference 0 mm, 95% CI: –26 to 34; p = 0.62). FIHOA scores were identical in both groups, with a median of 1.0 (range 0.1–2.2) in the isolated group and 1.0 (range 0.2–2.0) in the generalised group (median difference 0.0, 95% CI: –0.4 to 0.4; p = 0.99). HAQ scores were slightly higher in the generalised group (median 1.0, range 0.25–2.5) compared to the isolated group (median 0.75, range 0.125–2.0), but this difference was also not statistically significant (median difference 0.25, 95% CI: 0.0 to 0.5; p = 0.10).

Although these differences were not statistically significant at the p < 0.05 level, the trends align with previous findings suggesting that a broader OA phenotype carries increased functional burden.

In the isolated HOA group, a moderate positive correlation was observed between FIHOA and pain VAS (Spearman’s rho = 0.49, 95% CI: 0.17 to 0.71; p = 0.005) and between VAS and HAQ (rho = 0.37, 95% CI: 0.03 to 0.63; p = 0.039). In the generalised OA group, a strong positive correlation was found between FIHOA and VAS (rho = 0.75, 95% CI: 0.58 to 0.86; p < 0.001), while FIHOA and HAQ showed a weaker but significant correlation (rho = 0.32, 95% CI: 0.02 to 0.57; p = 0.034). The detailed results of these analyses are presented in **Table 4**.

**Table 4.** Correlation between FIHOA, VAS, and HAQ within Study Groups.

**Group**	**Variables**	**Spearman's rho**	**p-value**	**Interpretation**
Isolated HOA	FIHOA vs VAS pacienta (mm)	0.49	0.005	Significant
Isolated HOA	FIHOA vs HAQ	0.27	0.141	Not significant
Isolated HOA	VAS pacienta (mm) vs HAQ	0.37	0.039	Significant
Generalised OA	FIHOA vs VAS pacienta (mm)	0.75	0.0	Significant
Generalised OA	FIHOA vs HAQ	0.32	0.034	Significant
Generalised OA	VAS pacienta (mm) vs HAQ	0.26	0.083	Not significant

Spearman correlation coefficients between pain intensity (VAS), hand-specific function (FIHOA), and overall functional status (HAQ) in patients with isolated hand osteoarthritis (HOA) and generalised osteoarthritis (OA). Significant positive correlations indicate that higher pain levels are associated with greater functional impairment in both groups.

VAS: visual analog scale; FIHOA: Functional Index for Hand Osteoarthritis; HAQ: Health Assessment Questionnaire; HOA: hand osteoarthritis; OA: osteoarthritis.

## DISCUSSION

This study set out to determine whether HOA represents an isolated joint disorder or a clinical expression of generalised OA. Three key observations emerged. First, almost 60% of the cohort with radiographic HOA fulfilled criteria for OA at another site—most often the knees, spine and hips—confirming that HOA rarely occurs in true isolation. Second, patients with concomitant multi-joint OA reported numerically higher pain and disability on the VAS, FIHOA and HAQ instruments, even though the differences did not reach statistical significance after adjustment for sample size. Third, the distribution of additional joint involvement mirrored patterns described in large epidemiological datasets, with weight-bearing joints and the axial skeleton predominating.^10, 11^

### Relationship between HOA and systemic OA phenotypes

A growing body of evidence indicates that HOA frequently clusters with other OA phenotypes driven by shared biomechanical, metabolic and low-grade inflammatory mechanisms.^3,9,12^ In line with those reports, more than half of our HOA patients met the definition of generalised OA, and this subgroup carried a greater symptomatic burden. Although modest, the between-group differences in pain and disability corroborate the hypothesis that cumulative nociceptive input from multiple diseased joints amplifies the perception of pain and functional loss, thereby masking the “true” impact of HOA when assessed in isolation. Clinicians should therefore resist attributing the entirety of a patient’s disability to hand involvement alone, particularly in older women with metabolic risk factors—a constellation repeatedly linked to generalised OA phenotypes.^3,11^

In agreement with our findings, the European EPOSA study reported that hand OA is associated with functional limitations in older adults, independent of comorbidity burden.^13^ Similarly, the Nor-Hand longitudinal study confirmed that individuals with hand OA and higher comorbidity burden, including back pain and depression, experience more severe pain over time.^14^ Furthermore, the TLAR cohort from Turkey highlighted the association between hand OA and joint damage with functional limitations, supporting the view that HOA should be evaluated within the broader musculoskeletal context.^15^

Additionally, the observed significant correlations between pain intensity, hand-specific function (FIHOA), and global functional status (HAQ) in both patient groups suggest that higher pain levels are associated with greater functional impairment. This finding supports the idea that these indices reflect interrelated aspects of the symptomatic burden in HOA.

To our knowledge, this is the first study published in the Mediterranean Journal of Rheumatology that specifically examines the coexistence of HOA with OA in other joint regions and its impact on both pain and functional outcomes. While previous MJR publications have addressed various aspects of OA management and pathophysiology, none have focused on the clinical relevance of generalised OA phenotypes in the context of HOA. Our findings therefore add new insight into the importance of considering multi-joint involvement when assessing pain, disability, and management strategies in patients with hand OA.

### Interpretation of functional measures

The FIHOA and HAQ are validated instruments that focus on hand-centred activities of daily living.^5,8^ Our data reveal an important limitation: these tools underestimate disability attributable to lower-limb or spinal OA, as reflected by the relatively small HAQ difference despite clear multi-joint disease in Group B. Future studies should consider composite or whole-body functional indices—such as the AUSCAN or WOMAC scales—when global disease burden is the outcome of interest.

### Clinical implications

The finding that nearly two-thirds of HOA cases coexist with knee or spine OA has practical consequences:

- Diagnostic work-up: Routine radiographic or ultra-sonographic screening of symptomatic weight-bearing joints may uncover clinically relevant OA that would otherwise remain unrecognised.
- Management strategy: Non-pharmacological interventions (weight reduction, exercise therapy) and systemic pharmacological options (e.g. slow-acting symptomatic drugs) should be prioritised over purely local hand-directed treatments in this phenotype.
- Prognosis and counselling: Patients with multi-joint OA can be counselled about the higher likelihood of progressive disability and the potential need for multidisciplinary care, including physiotherapy targeting lower-limb strength and spinal flexibility.

### Strengths and Limitations

A key strength of this study is the strict radiographic inclusion threshold (Kellgren–Lawrence ≥ 2), which reduces the risk of misclassification of early or doubtful osteoarthritis. In addition, the dataset incorporates validated patient-reported outcome measures (FIHOA, HAQ, VAS) that capture both pain and functional status in this population.

Several limitations should be acknowledged. First, the retrospective observational design precludes the establishment of temporal or causal relationships and limits the ability to assess disease progression over time. Second, although the sample size is typical for single-centre HOA studies, it limited the statistical power to detect small but clinically meaningful differences between groups. Third, reliance on clinical examination rather than standardised imaging for non-hand joints may have led to underestimation of the prevalence and severity of generalised osteoarthritis. Finally, the absence of biochemical and metabolic parameters restricted exploration of systemic OA phenotypes such as metabolic OA.

### Future directions

Prospective, multicentre cohorts with longitudinal imaging and systemic biomarker panels are required to delineate shared pathophysiological pathways linking hand, knee, hip and spinal OA. Such studies will clarify whether the “generalised” phenotype is a distinct nosological entity or the cumulative result of joint-specific biomechanical insults superimposed on systemic susceptibility factors.

Collectively, our data reinforce the concept that HOA is frequently part of a wider osteoarthritic constellation rather than an isolated hand disorder. Recognition of multi-joint involvement is essential for accurate assessment of pain and disability and should inform a comprehensive, patient-centred management plan that extends beyond the hands.

## CONCLUSION

In this study, patients with hand osteoarthritis and additional OA in other joint sites showed slightly higher levels of pain and functional impairment than those with isolated HOA. These results suggest that HOA frequently coexists with generalised OA and that non-hand joint involvement may contribute to overall disability. Clinicians should consider evaluating for generalised OA when assessing patients with hand symptoms, as this may influence both prognosis and therapeutic decisions.

Our findings are consistent with prior research showing that multi-joint OA is associated with increased levels of disability and pain, regardless of which joint is initially affected.^11,13,15^

Notably, studies have highlighted that OA phenotypes often overlap, and the coexistence of hand OA with lower limb OA may represent a more severe or systemic form of the disease.^12,16^

Recent investigations have emphasised the importance of including global musculoskeletal assessment in patients with hand OA to avoid underestimating the total burden of disease.^17^

From a practical perspective, this supports the integration of multidisciplinary care and broader rehabilitation approaches for patients presenting with HOA and co-morbid OA features.

## DATA SHARING STATEMENT

The datasets generated and/or analysed during the current study are not publicly available due to patient confidentiality and institutional regulations but are available from the corresponding author on reasonable request.

## PRESENTATION AT SCIENTIFIC MEETINGS

Preliminary results of this study were presented in oral form under the title “Fyzická disabilita a komorbidity u pacientov so symptomatickou osteoartrózou rúk” by Veronika Farská, Ivan Rybár, and Pavol Masaryk at the 65th Congress of Slovak and Czech Rheumatologists in conjunction with the 25th Congress of the Slovak Medical Society, held in Bratislava, Slovakia, from November 30 to December 1, 2022.
